# The Golgin Tether Giantin Regulates the Secretory Pathway by Controlling Stack Organization within Golgi Apparatus

**DOI:** 10.1371/journal.pone.0059821

**Published:** 2013-03-21

**Authors:** Mayuko Koreishi, Thomas J. Gniadek, Sidney Yu, Junko Masuda, Yasuko Honjo, Ayano Satoh

**Affiliations:** 1 The Graduate School of Natural Science and Technology, Okayama University, Okayama, Japan; 2 Department of Pathology, Johns Hopkins University School of Medicine, Baltimore, Maryland, United States of America; 3 School of Biomedical Sciences and Epithelial Cell Biology Research Center, The Chinese University of Hong Kong, Shatin, N.T., Hong Kong SAR, People’s Republic of China; 4 Mucosal Immunity Section, Laboratory of Host Defenses, National Institute of Allergy and Infectious Diseases, National Institutes of Health, Bethesda, Maryland, United States of America; 5 The Research Core for Interdisciplinary Sciences (RCIS), Okayama University, Okayama, Japan; Institut Jacque Monod, Centre National de la Recherche Scientifique, France

## Abstract

Golgins are coiled-coil proteins that play a key role in the regulation of Golgi architecture and function. Giantin, the largest golgin in mammals, forms a complex with p115, rab1, GM130, and soluble N-ethylmaleimide-sensitive factor attachment protein receptors (SNAREs), thereby facilitating vesicle tethering and fusion processes around the Golgi apparatus. Treatment with the microtubule destabilizing drug nocodazole transforms the Golgi ribbon into individual Golgi stacks. Here we show that siRNA-mediated depletion of giantin resulted in more dispersed Golgi stacks after nocodazole treatment than by control treatment, without changing the average cisternal length. Furthermore, depletion of giantin caused an increase in cargo transport that was associated with altered cell surface protein glycosylation. Drosophila S2 cells are known to have dispersed Golgi stacks and no giantin homolog. The exogenous expression of mammalian giantin cDNA in S2 cells resulted in clustered Golgi stacks, similar to the Golgi ribbon in mammalian cells. These results suggest that the spatial organization of the Golgi ribbon is mediated by giantin, which also plays a role in cargo transport and sugar modifications.

## Introduction

The majority of intracellular membrane traffic involves the formation, transport, and selective fusion of membrane-bound vesicles. To bud from a donor membrane, vesicles generally require coat proteins that induce membrane curvature. Well-studied coat protein complexes include coatomer protein I (COPI) within the Golgi apparatus, coatomer protein II (COPII) at endoplasmic reticulum exit-sites, and clathrin at the trans-Golgi and cell surface membrane [1], [2], [3].

For accurate vesicular transport, vesicles must dock and fuse with their proper target membrane, which involves coordinated and specific protein-protein interactions. For example, the targeting of COPI vesicles is considered to be a multi-layered process that requires Rab\Arl GTPases, tethers, and soluble N-ethylmaleimide-sensitive factor attachment protein receptors (SNAREs) [4]. Tethers include coiled-coil proteins of the golgin family and multi-protein complexes, such as TRAPP and COG [5], [6], [7]. Once a vesicle is tethered to its target membrane, vesicle docking and membrane fusion are mediated by SNAREs and accessory proteins [8]. SNARE proteins contain evolutionarily conserved 60–70 amino acid SNARE motifs arranged in heptad repeats that confer specificity to vesicle membrane (v-SNAREs) and target membrane (t-SNARE) interactions. The asymmetric distribution of v-SNAREs and t-SNAREs among intracellular membrane compartments allows for specific vesicle and target membrane fusion events. However, the earliest stage of SNARE pairing between membranes cannot occur at distances of more than 25 nm, suggesting that tethers play an important role in the initial contact between vesicles and their target membrane. Along these lines, we have previously shown that golgin tethers play a role in specifying vesicle fusion sites within the Golgi apparatus [9].

Golgins are a family of coiled-coil proteins that are anchored either directly, via a membrane spanning domain, or indirectly, through interactions with other golgins or Rab/Arl GTPases [5]. Specific golgins are found at different locations within the Golgi apparatus, where they organize Golgi and cisternal stacking and tether COPI vesicles to the Golgi membranes [5].

The “cis-golgin tether” is one of the most well-characterized golgin tether complexes. It is composed of the COPI vesicle-associated golgin giantin linked to Golgi membrane-associated GM130 via p115. GM130 is in turn linked to GRASP65 via a PDZ-like domain. GRASP65 is anchored to the Golgi membrane through N-terminal myristoylation as well as through binding to other Golgi proteins [10]. Together, these proteins appear to mediate vesicle tethering at the cis-Golgi membrane.

We have also identified a new golgin tether consisting of the COPI vesicle-associated protein golgin-84 and the Golgi membrane-associated protein CASP. It appears that COPI vesicles tethered by the golgin-84/CASP are involved in Golgi enzyme transport, whereas COPI vesicles tethered by the cis-golgin tether giantin/GM130 are involved in cargo transport [9]. Interestingly, COPI vesicles utilizing golgin-84/CASP tethers lack anterograde cargo and p24 family proteins, which are putative cargo receptors within COPI vesicles. In contrast, COPI vesicles utilizing the giantin/GM130 tethering complex are enriched for anterograde cargo and the p24 family of cargo receptor proteins [9]. Taken together, these results suggest that these two different golgin tether complexes may define functionally distinct sub-populations of COPI vesicles. Conceptually, we propose that giantin and golgin-84 should be considered vesicle-associated tethers (v-ATs), similar to v-SNAREs.

Giantin is the largest identified golgin protein [5]. Previous studies have demonstrated that giantin consists of a transmembrane domain with either a short or no luminal domain at its C terminus, depending on the species [11]. Giantin binds directly to the C terminus of p115 [12], [13] to form part of the cis-golgin tether. Although the contribution of Rab1 to cis-golgin tether formation is currently unclear, giantin has been shown to bind directly to Rab1 [14], [15]. We have proposed a model wherein giantin and GM130 facilitate p115-mediated recruitment of rab1 to Golgi membranes, since giantin and GM130 stimulate p115 binding to Rab1 [14]. In addition to members of the cis-golgin tether, giantin has several other binding partners, including Rab6 and GCP60 [15], [16]. Given its large size, giantin could potentially interact simultaneously with its various binding partners.

## Results

### Giantin Localized to COPI Vesicles and Golgi Buds and Cisternal Rims

**Figure 1:**
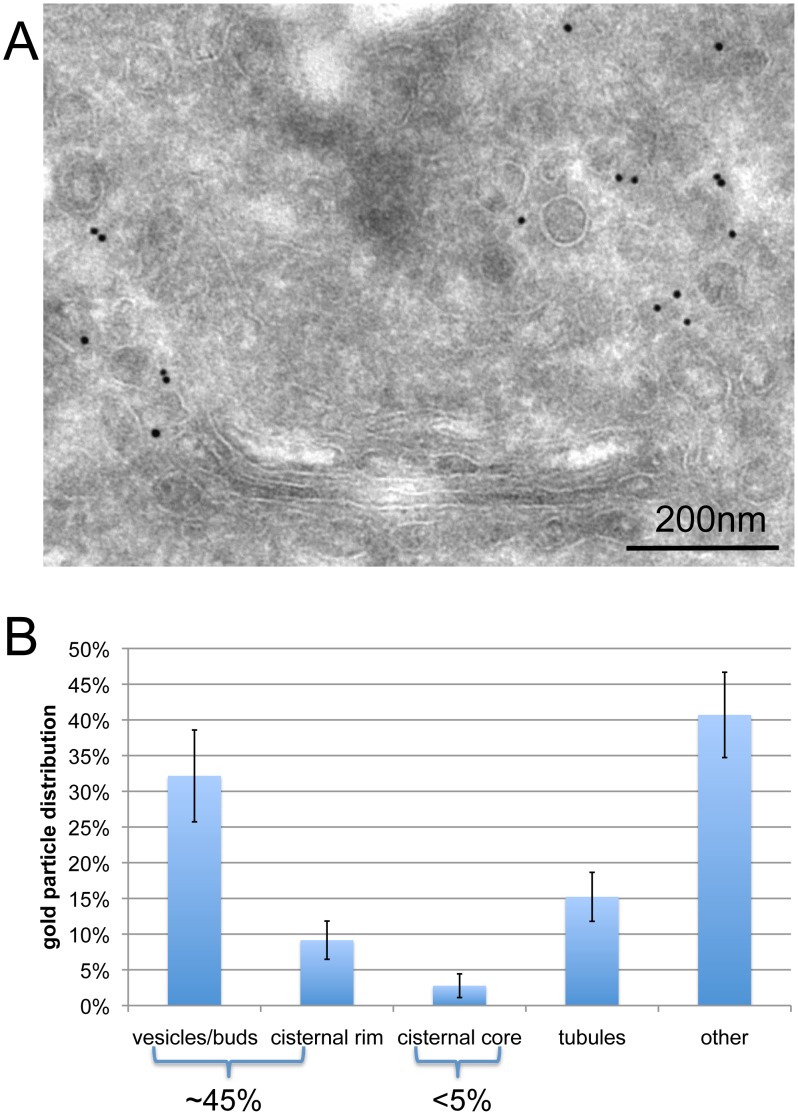
Giantin localized to buds, vesicles, and cisternal rims. (A) HeLa cells were processed for cryo-electron microscopy and immunolabeled for giantin. Bar, 200 nm. (B) Quantitation shows that giantin was localized primarily to buds, vesicles, and cisternal rims.

To gain additional insight into the function of giantin, we analyzed the localization of giantin by immunoelectron microscopy (immune-EM) using anti-giantin pAb. As shown in Figure 1A and quantitated in Figure 1B, approximately 45% of giantin was localized to vesicles, Golgi buds, and Golgi cisternal rims. This is in agreement with previously reported biochemical and electron microscopy data, which show that giantin is found in COPI vesicles [16]. In addition, giantin was found in all compartments in the Golgi cisternae, unlike GM130, whose localization is highly restricted to cis-Golgi cisternae [17], [18], [19], [20], [21]. This finding suggests that giantin may have another function in addition to its role in the cis-golgin tether, wherein giantin resides on COPI vesicles and is linked via a soluble protein, such as p115, to GM130 on Golgi cisternae for COPI vesicle tethering [10].

### Nocodazole Treatment of Giantin RNAi Cells Resulted Smaller Golgi Stacks

**Figure 2:**
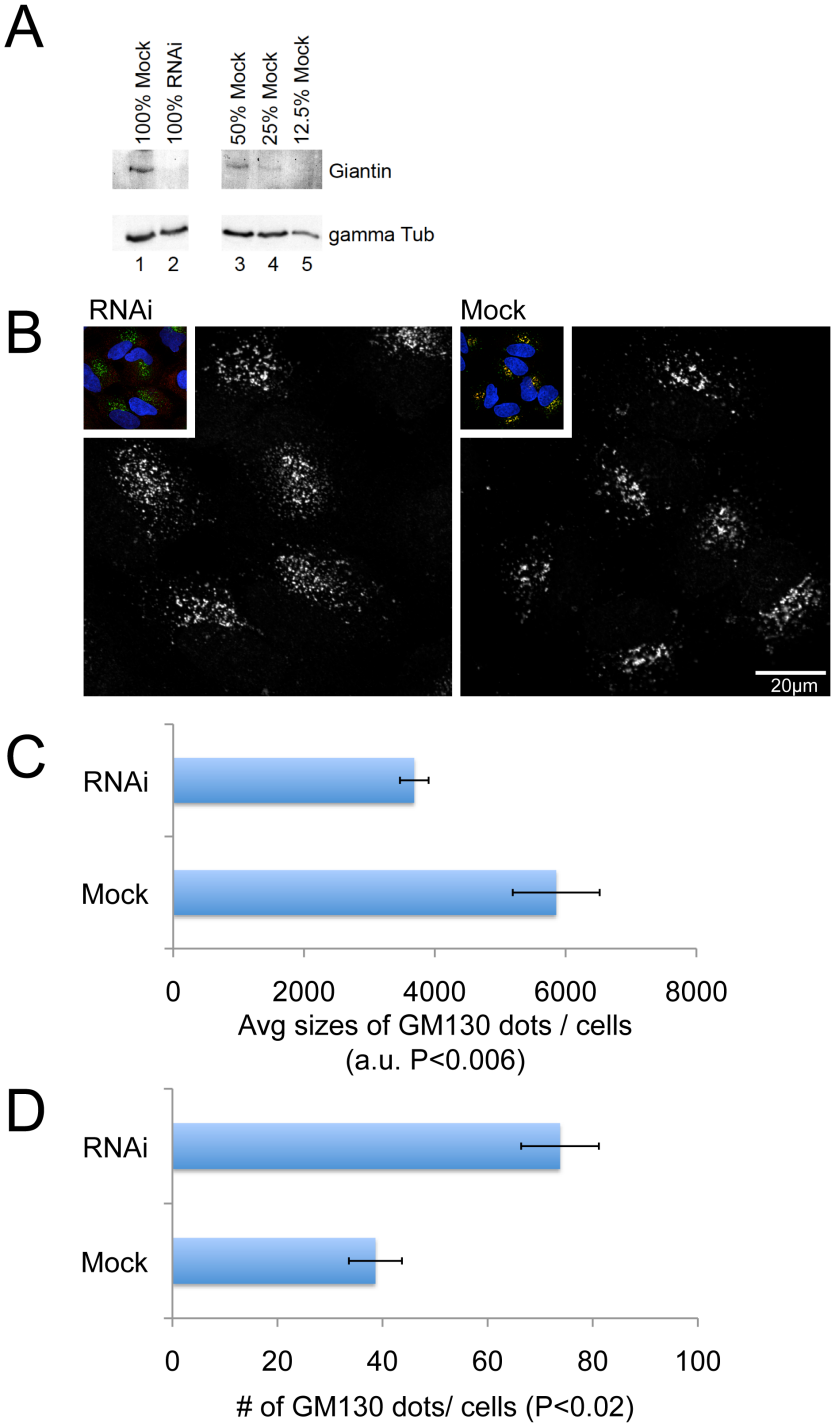
Giantin RNAi dispersed Golgi mini-stacks generated by nocodazole treatment. (A) Equal amounts (lanes 1 and 2) and serial dilutions (lanes 3–5) of total cell lysates from giantin siRNA- and mock-treated cells were loaded and subjected to immunoblotting to determine the degree of knockdown; giantin (upper panel), gamma tubulin (lower panel). Giantin protein levels were reduced by 85%–95% in all giantin siRNA-treated cells in the experiments presented herein. Giantin siRNA- and mock-treated cells were incubated with nocodazole (0.2 µg/ml) for 45 min, and then subjected to indirect immunofluorescence for gianitn (red), GM130 (green), and nuclei (blue). GM130 staining is shown in (B). Insets in (B) are the merged images of all three colors. Normal sized cells were selected and quantified; the sizes of GM130-positive dots are shown (C). Bars in (C) represent SD of the average sizes of GM130-positive dots in a cell (n = ∼20 cells).

Given giantin’s localization to various areas of the Golgi complex, we examined whether depletion of giantin would influence the morphology of the Golgi apparatus and the rate of intracellular membrane protein traffic. In order to do this, we performed siRNA-mediated giantin RNAi. To assess the efficiency of giantin RNAi, giantin protein levels in serial dilutions of mock- and siRNA-treated cells were compared by western blotting. As shown in Figure 2A, giantin protein levels were reduced by more than 87% in giantin siRNA-treated cells. Of note, giantin protein levels were reduced by 85–95% in all of the experiments performed herein. Although giantin was almost completely depleted in giantin siRNA-treated cells, we did not observe obvious morphological changes in the Golgi apparatus by immunofluorescence (data not shown). However, when these giantin-depleted cells were treated with nocodazole, a microtubule-depolymerizing agent known to transform Golgi ribbons into Golgi stacks, Golgi stacks in giantin siRNA-treated cells appeared more dispersed when compared to cells treated with nocodazole alone (Figure 2B). As shown in the quantitation in Figure 2C and 2D, after nocodazole treatment, giantin siRNA decreased the average Golgi stack size and increased the average number of Golgi, each by approximately 60% compared to mock siRNA control cells. This alteration in the size and number of Golgi stacks within each cell suggests that Giantin may play a role in organizing and/or stabilizing Golgi stacks. This phenotype was observed when a different giantin siRNA was used and was reversed by an exogenous expression of rat giantin cDNA (siRNA2, Figure S1B).

**Figure 3:**
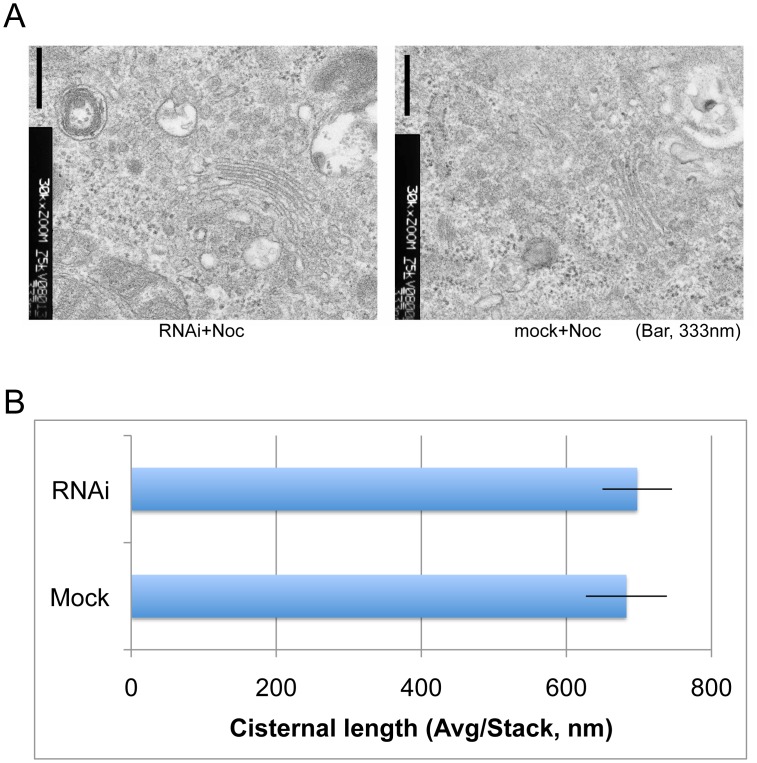
Giantin RNAi did not change the cisternal lengths of Golgi mini-stacks generated by nocodazole treatment. (A) Nocodazole, giantin siRNA-, and mock-treated cells were processed for electron microscopy. Bar, 333 nm. (B) Average length of Golgi cisternae in a Golgi stack in approximately 10 different cells. Bars represent SEM (n = ∼10 cells).

Since the depletion of giantin influenced the organization of Golgi stacks in nocodazole-treated cells, we examined if the depletion of giantin changes the length of Golgi cisternae. Mock and giantin siRNA- nocodazole-treated cells shown in Figure 2B were processed for EM. As shown in Figure 3A and quantitated in Figure 3B, no significant difference was observed in the appearance of Golgi cisternae, quantitated by the average cisternal length per stack. Therefore, both EM (Figure 3) and immunofluorescence (Figure 2) data support the concept that giantin may be involved in the organization of Golgi stacks.

### Exogenous Expression of Giantin in Drosophila S2 Cells Organized their Dispersed Golgi Stacks

**Figure 4:**
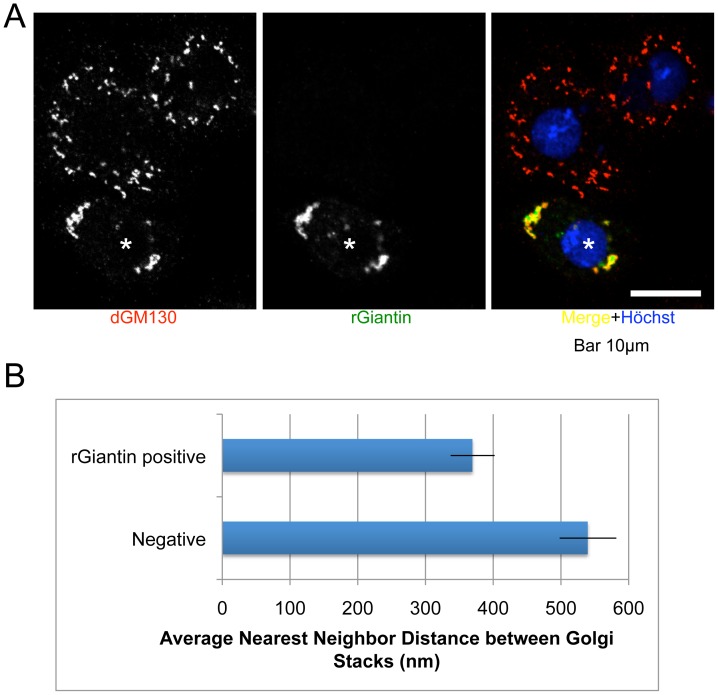
Exogenous expression of giantin in Drosophila S2 cells organized their dispersed stacks. (A) Rat giantin cDNA was transiently transfected into S2 cells. The cells were processed for immunofluorescence imaging of dGM130 (Golgi marker, in red), rat giantin (in green), and nuclei (blue). The asterisk marks the rat giantin-expressing cell. Bar, 10 nm. (B) Dispersion analysis was performed by measuring the average nearest neighbor distance between segmented GM130-positive Golgi stacks with or without rat Giantin expression.

Since nocodazole and giantin siRNA-treated cells exhibited more dispersed Golgi stacks, we hypothesized that giantin may be involved in the organization of Golgi stacks. Many mammalian cells are known to have organized Golgi stacks, called Golgi ribbons, whereas invertebrate cells, e.g., *Drosophila* cells, which do not express an apparent giantin homologue, have dispersed Golgi stacks instead of Golgi ribbons. Therefore, we used the S2 cell line, derived from *Drosophila,* to examine the function of giantin in the organization of Golgi stacks. As shown in Figure 4A, transient expression of rat giantin cDNA in S2 cells partially organized Golgi stacks. To quantitate the organization and dispersion of Golgi stacks, dispersion analysis was performed using a custom Image J plug-in. Cells were manually selected for analysis and the GM130 signal was smoothed with a mean filter (radius 2 pixels). Local maxima were found within the resulting data and the list of local maxima was thresholded using Image J’s build-in IsoData thresholding algorithm. Finally, the average nearest neighbor distance was calculated for each cell and tabulated as a measure of dispersion. As shown in Figure 4B, the average nearest neighbor distance between Golgi stacks is 70% shorter in S2 cells highly expressing rat Giantin (n = ∼20, P<0.01). These data strongly support the concept that giantin is involved in the organization of Golgi stacks.

### Giantin RNAi Affected Anterograde Transport and Surface Glycosylation Patterns

**Figure 5:**
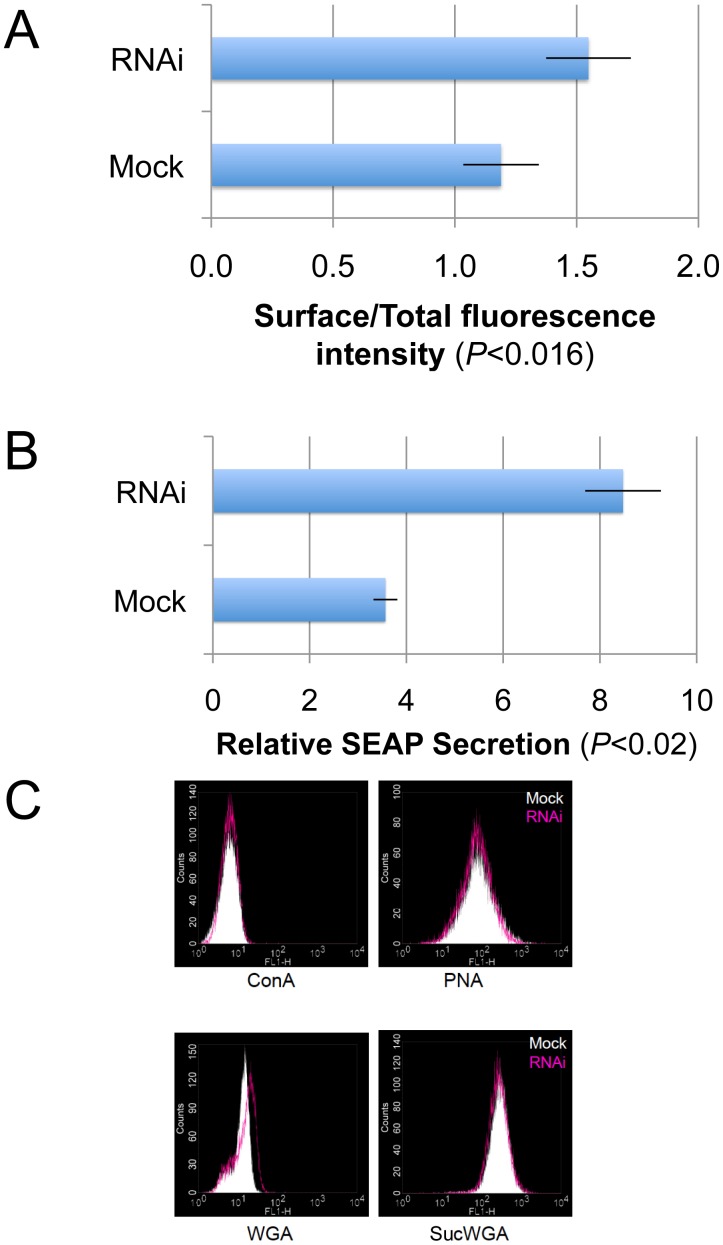
Giantin RNAi enhanced anterograde trafficking and changed surface glycosylation patterns. (A) Giantin siRNA- and mock-treated cells were transfected with VSV-G-tsO45-YFP and incubated at the restrictive temperature of 40°C overnight before shifting them to the permissive temperature of 32°C. Cells were surface labeled for VSV-G. Cell images were captured and analyzed using Image J and Photoshop (n = ∼20 cells). Error bars represent SEM of three independent experiments. (B) SEAP was increased by giantin RNAi. HeLa cells stably expressing SEAP were transfected with siRNAs. After 90 h, the cells were washed and fresh culture media were added. After 6 and 24 h, respectively, culture supernatants were collected and processed for SEAP activity measurement. The ratio of the activities after 6 and 24 h are shown in the graph. Bars, SD (n = 3). (C) Giantin siRNA- or mock-treated cells were surface-labeled with FITC-lectins and analyzed by flow cytometry. SucWGA does not bind to sialyl-sugar moieties, unlike the native form, but retains its specificity to N-acetylglucosamine.

To determine whether giantin RNAi influences trafficking, we performed a Vesicular stomatitis virus G protein (VSV-G) transport assay. VSV-G-tsO45 is a temperature-sensitive mutant of the G protein from vesicular stomatitis virus. It has been used widely for transport assays because it accumulates in the ER at a restrictive temperature of 40°C and moves to plasma membranes after a shift to the permissive temperature of 32°C. As shown in Figure 5A, plasma membrane-associated VSV-G-tsO45 increased upon treatment with giantin siRNA. The secretion of another anterograde cargo, secretory alkaline phosphatase (SEAP) was increased more than twice by treatment with giantin siRNA (Figure 5B). This phenotype was also observed when other giantin siRNAs were used and was reversed by exogenous expression of rat giantin cDNA (siRNA2, Figure S1C).

To gain insights into giantin RNAi-associated alterations in anterograde transport, we used lectin staining to examine cell surface glycosylation patterns. Since gylcosylation occurs mainly in the Golgi apparatus, changes in the cell surface glycosylation pattern may reflect changes in glycosylation in the Golgi apparatus. The sugar-binding specificities of lectins used herein include Con A (Concanavalin A), terminal alpha-linked mannose; peanut agglutinin (PNA), galactosyl (beta-1,3) N-acetylgalactosamine; wheat germ agglutinin (WGA), N-acetylglucosamine, and sialic acids on glycoproteins. It is known that succinylated WGA (SucWGA) does not bind to sialic acid, unlike the native form, but retains its specificity toward N-acetylglucosamine. Therefore, the use of native WGA and succinylated form can distinguish between sialylated glycoconjugates and those containing only N-acetylglucosamine structures. As shown in Figure 5C, giantin siRNA- and mock-treated cells exhibited similar surface expression patterns of Con A-, PNA- and SucWGA-reactive glycans. However, the WGA-reactive glycans were slightly but significantly increased in giantin siRNA-treated cells, suggesting that giantin affected the surface expression of sialyl glycans.

## Discussion

Given the cis-golgin tether model, one might predict that depletion of giantin would lead to the aberrant trafficking of Golgi proteins into untethered cytoplasmic COPI vesicles. However, this was not observed in our study. Instead, we found that the organization of Golgi stacks in nocodazole-treated cells was disrupted upon depletion of giantin (Figure 2) without changing the cisternal lengths of the Golgi stacks (Figure 3). In particular, the nocodazole fragmented Golgi ministacks were further dispersed by depletion of giantin. Furthermore, exogenous expression of giantin cDNA in Drosophila cells induced a relative clustering of their normally dispersed Golgi stacks, supporting the notion that giantin plays a role in Golgi organization (Figure 4, summarized in Figure S2).

One possible interpretation of this data is that giantin depletion disrupted Golgi stack clustering. The fact that the length of nocodazole treated Golgi ministacks did not change after giantin depletion suggests that lateral Golgi ministack fragmentation did not occur. Therefore, the Golgi dispersion induced by giantin depletion is likely due to decreased stack clustering, leading to more and smaller Golgi stacks when counted by a global thresholding method (Figure 2).

Giantin may mediate Golgi stack clustering via lateral membrane trafficking between Golgi stacks. Previous work has shown that lateral trafficking is necessary for Golgi stack biogenesis [22], [23]. If true, this may explain an observed increase in trafficking of anterograde cargo in Giantin depleted cells, since inhibiting lateral trafficking may increase the flux through forward trafficking pathways. To address, this possibility, further experiments involving model organisms that contain simple Golgi architecture, such as *T. brucei,* may be warranted. Another possible mechanism for giantin mediated Golgi stack clustering involves direct molecular interactions from one Golgi stack to another. To test such a hypothesis further experiments may seek to precisely measure the minimum inter-Golgi stack distance. If this distance is highly consistent, it would argue in favor of a fixed-length direct connection.

Finally, our data showed an increased rate of trafficking of anterograde cargo associated with alterations of cell surface glycosylation patterns in giantin-depleted cells (Figure 5). One could imagine that the increase in WGA-reactive glycans in giantin-depleted cells indicates an increase in trans-Golgi located terminal sugar modification (sialylation), which is likely to cause slower protein trafficking through the sialyltransferase-containing compartment. However, our results did not support this concept. It would be interesting to analyze the correlation between glycosylation patterns and trafficking in future. Additionally, it is unclear whether the absence of giantin in Drosophila confers an evolutionary advantage via alterations to its glycosylation pathways. Therefore, future research will seek to investigate the mechanism by which changes in trafficking and Golgi organization alter the sugar modification of glycoproteins and glycolipids.

## Materials and Methods

### Antibodies and Lectins

Monoclonal anti-GM130 (BD Biosciences, San Jose, CA), polyclonal anti-giantin ([24], and ab24586; Abcam, Cambridge, MA), monoclonal anti-giantin [(Dr. Adam Linstedt (Carnegie Mellon), anti-gamma tubulin (GTU-88; Sigma, St. Louis, MO), anti-dGM130 (ab30637, Abcam), and anti-VSV-G (VG; Dr. Ira Mellman (Genentech)] were the antibodies used in this study. All FITC-labeled lectins and HRP-conjugated secondary antibodies were purchased from Vector Laboratories (Burlingame, CA) and Pierce (Rockford, IL), respectively. All Alexa-labeled secondary antibodies and Hoechst were purchased from Invitrogen (Carlsbad, CA).

### Plasmids and siRNAs

VSV-G-tsO45-SP-YFP (VSV-G-YFP) and FLAG-tagged rat giantin were provided by Dr. Derek Toomre (Yale University, CT) and Dr. Yoshio Misumi (Fukuoka University, Fukuoka, Japan) [25], [26], respectively. Giantin cDNA from rats was subcloned into the pMT/V5-His A vector (Invitrogen, Carlsbad, CA) for expression in S2 cells. Giantin siRNAs [27] and eGFP (mock RNAi) were purchased from Dharmacon (Lafayette, CO), Santa Cruz (Santa Cruz, CA), and Qiagen (Valencia, CA). siRNA1∶5′-ACUUCAUGCGAAGGCCAAA-3′; siRNA2∶5′-CUGGAGUAGAAUUGAAAUCAA-3′.

### Cell Culture and Transfection

HeLa cells (CCL-2, ATCC, Manassas, VA) were grown in Dulbecco’s modified Eagle’s medium supplemented with 10% FBS (Invitrogen, Carlsbad, CA) at 37°C. Transfection of HeLa cells with plasmids and siRNAs was performed using Lipofectamine LTX (Invitrogen) and RNAiMAX (Invitrogen), respectively, according to the manufacturer’s instructions. For depolymerization of microtubules, cells were treated with nocodazole (0.2 µg/ml) (Sigma, St Louis, MO) for 30–50 min [28].

S2 cells (RCB1153, RIKEN BRC through the National Bio-Resource Project of the MEXT, Tsukuba, Ibaragi, Japan) were grown in S2 cell medium supplemented with 10% FBS (Invitrogen, Carlsbad, CA) at 28°C. Transfection of S2 cells with plasmids was performed by the calcium phosphate method, with subsequent induction of expression by CuSO_4_, according to the manufacturer’s instruction (Invitrogen).

### Immunofluorescence Microscopy

HeLa cells grown on coverslips were fixed with 3.7% PFA in PBS for 15 min, and permeabilized with 0.1% Triton X-100 in PBS for 5 min at room temperature. Next, the cells were blocked with 4% BSA in PBS for 15 min, and incubated for 15 min with primary antibodies diluted in 4% BSA in PBS. The cells were washed three times with PBS, and incubated for 15 min with secondary antibodies conjugated to Alexa fluorophores (Invitrogen). After washing the cells, coverslips were mounted on microscope slides and viewed using an FV1000 confocal microscope (Olympus, Tokyo, Japan). For immunofluorescence of S2 cells, cells were stripped from the culture dish by gentle pipetting and placed on 0.5 mg/ml Con A-treated coverslips [29]. After incubation for 1 h at 28°C, the coverslips were treated as described above. Images were quantified using the Image J program.

### VSV-G Transport Assay

The VSV-G transport assay was performed as previously described [9], [30]. In brief, HeLa cells were transfected with siRNA. After 72 h, cells were transfected again with the VSV-G-YFP plasmid and incubated at the restricted temperature of 40°C overnight. After shifting to the permissive temperature of 32°C, cells were incubated for 90 min and processed for immunofluorescence to label cell surface VSV-G.

### SEAP Transport Assay

The SEAP transport assay was performed as previously described [31]. In brief, HeLa cells stably expressing SEAP were transfected with siRNAs. After 90 h, the cells were washed and fresh culture media were added. After 6 and 24 h, respectively, culture supernatants were collected and processed for SEAP activity measurement using the Phospha-light system (Applied Biosystems). Data are presented as a secretion index, which is the ratio of SEAP activity detected in the culture supernatant at 6 h to that detected at 24 h.

### Electron Microscopy

Immunogold labeling with anti-giantin and electron microscopy were performed as previously described [9], [20] at the Yale University Cell Biology and Okayama University Medical School EM core facilities.

### FACS Analysis

FACS analysis was performed as previously described [32]. In brief, cells were detached from culture dishes by short trypsin-EDTA treatment, and then blocked with PBS containing 0.2% FBS for 30 min. After blocking, cells were incubated with fluorescein-labeled lectins (Vector Laboratories) for 30 min on ice. After washing, cells were analyzed by FACS Calibur (BD Biosciences).

### Dispersion Analysis

To quantitate the organization and dispersion of Golgi stacks, dispersion analysis was performed using a custom ImageJ plug-in. Cells were manually selected for analysis and the GM130 signal was smoothed with a mean filter (radius = 2). Local maxima were found within the resulting data and thresholded using ImageJ’s build-in IsoData thresholding algorithm [32] to remove background local maxima. Finally, the average nearest neighbor distance [33] was calculated for each cell and tabulated as a measure of dispersion.

## Supporting Information

Figure S1 **Other human giantin siRNA (siRNA2) also caused the giantin RNAi phenotype that was reversed by an exogenous expression of rat giantin.** (A) Equal amounts of total cell lysate from siRNA2- and mock-treated cells were loaded, and then subjected to immunoblotting to determine the degree of giantin knockdown; giantin (upper panel), gamma tubulin (lower panel). Giantin siRNA2 reduced giantin protein levels by approximately 80%. (B) HeLa cells were transfected with or without giantin siRNA2. After 72 h, a rat giantin expression plasmid was transfected into one batch of siRNA2-transfected cells. After a further 24 h of incubation, the cells were incubated with nocodazole (0.2 µg/ml) for 45 min, and then subjected to indirect immunofluorescence as described in Figure 2. Cells with normal sizes were selected and quantified; the average sizes of GM130-positive dots are shown. Bars represent SD (n = ∼20 cells). (C) SEAP was increased by siRNA2. HeLa cells stably expressing SEAP cDNA were transfected with or without giantin siRNA2. After 72 h, rat giantin cDNA was transfected to one batch of siRNA2-transfected cells. After a further 24 h, cells were washed, aliquots of culture supernatants were collected after another 6 and 24 h, and then phosphatase activities were measured as described in Figure 5. The ratio of the activities 6 and 24 h after washing are shown in the graph. Bars represent SD (n = 3). Inset in (C) shows giantin protein levels in the samples obtained by western blotting. Giantin protein levels in the samples were normalized using gamma tubulin levels and their ratios are presented below.
(TIF)

Figure S2 **Working model.** The mammalian cell Golgi ribbon (upper diagram) is known to be fragmented by Nocodazole, a microtubule-disrupting agent, and transformed into separated ministacks. We show that Nocodazole fragmented Golgi ministacks are further dispersed by the depletion of Giantin. Giantin’s contribution to Golgi ministack aggregation may be through direct inter-stack bridging or vesicular trafficking.
(TIF)
