# Supplementary figures and images for: The Golgin Tether Giantin Regulates the Secretory Pathway by Controlling Stack Organization within Golgi Apparatus
Source: PLoS One. 2013 Mar 21;8(3):e59821. doi: 10.1371/journal.pone.0059821 (PMC3605407; doi:10.1371/journal.pone.0059821)

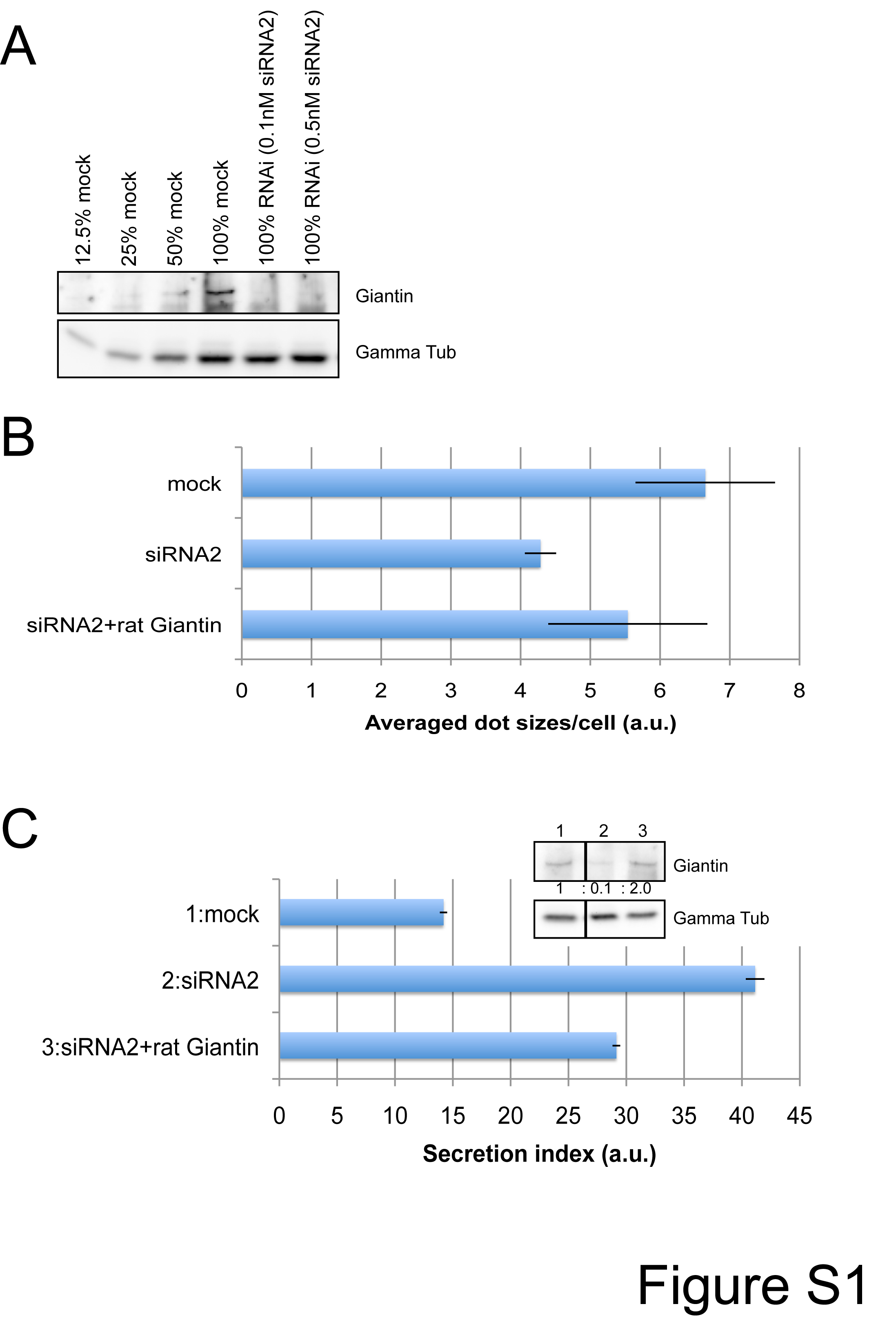

Supplement: Figure S1 — Other human giantin siRNA (siRNA2) also caused the giantin RNAi phenotype that was reversed by an exogenous expression of rat giantin. (A) Equal amounts of total cell lysate from siRNA2- and mock-treated cells were loaded, and then subjected to immunoblotting to determine the degree of giantin knockdown; giantin (upper panel), gamma tubulin (lower panel). Giantin siRNA2 reduced giantin protein levels by approximately 80%. (B) HeLa cells were transfected with or without giantin siRNA2. After 72 h, a rat giantin expression plasmid was transfected into one batch of siRNA2-transfected cells. After a further 24 h of incubation, the cells were incubated with nocodazole (0.2 µg/ml) for 45 min, and then subjected to indirect immunofluorescence as described in Figure 2. Cells with normal sizes were selected and quantified; the average sizes of GM130-positive dots are shown. Bars represent SD (n = ∼20 cells). (C) SEAP was increased by siRNA2. HeLa cells stably expressing SEAP cDNA were transfected with or without giantin siRNA2. After 72 h, rat giantin cDNA was transfected to one batch of siRNA2-transfected cells. After a further 24 h, cells were washed, aliquots of culture supernatants were collected after another 6 and 24 h, and then phosphatase activities were measured as described in Figure 5. The ratio of the activities 6 and 24 h after washing are shown in the graph. Bars represent SD (n = 3). Inset in (C) shows giantin protein levels in the samples obtained by western blotting. Giantin protein levels in the samples were normalized using gamma tubulin levels and their ratios are presented below. (TIF) [file pone.0059821.s001.tif]

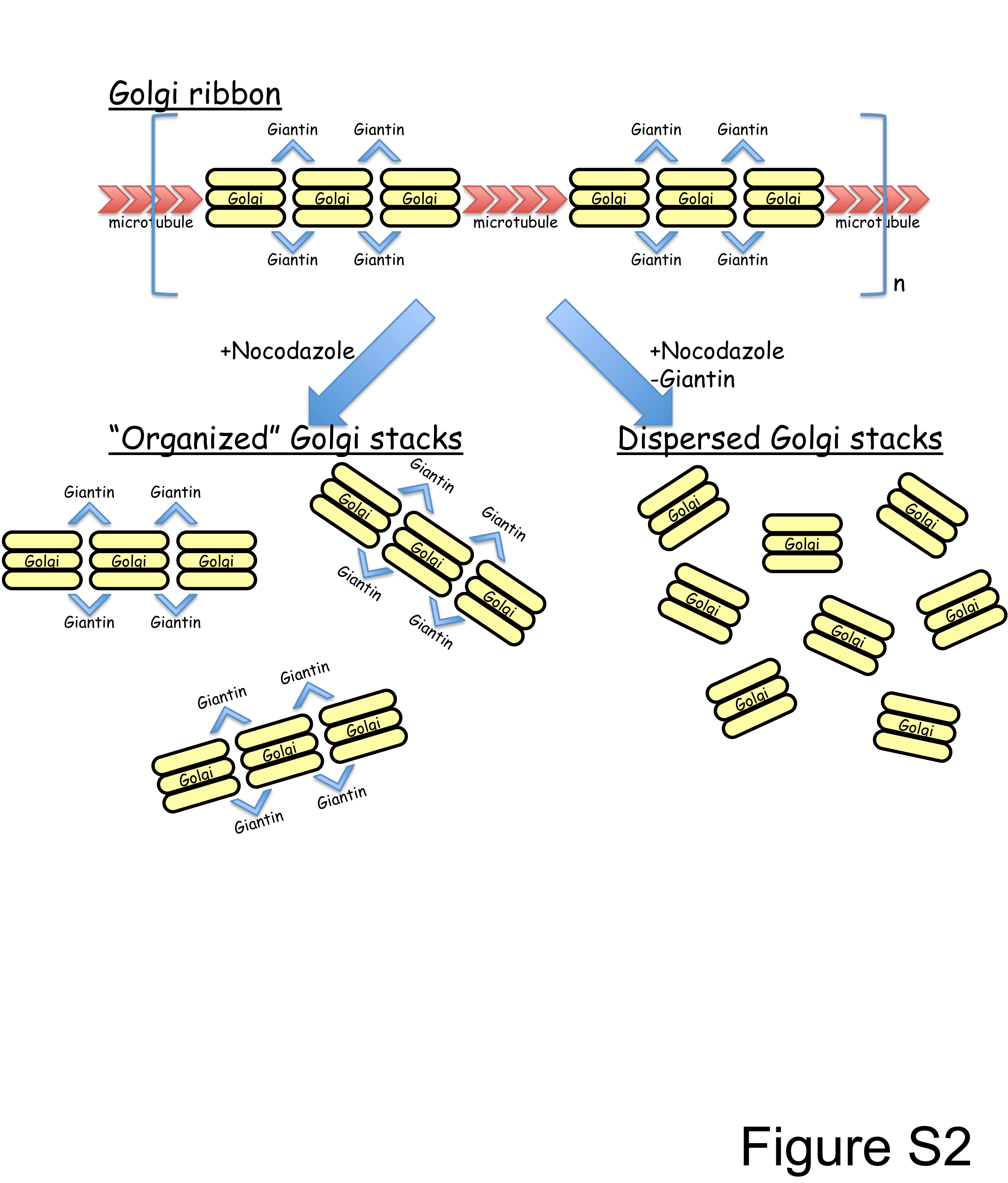

Supplement: Figure S2 — Working model. The mammalian cell Golgi ribbon (upper diagram) is known to be fragmented by Nocodazole, a microtubule-disrupting agent, and transformed into separated ministacks. We show that Nocodazole fragmented Golgi ministacks are further dispersed by the depletion of Giantin. Giantin’s contribution to Golgi ministack aggregation may be through direct inter-stack bridging or vesicular trafficking. (TIF) [file pone.0059821.s002.tif]
